# Bowel frequency (night) and urgent defecation are improved by budesonide foam in patients with ulcerative colitis: a retrospective observational study

**DOI:** 10.1186/s12876-022-02388-6

**Published:** 2022-06-24

**Authors:** Ryosuke Miyazaki, Toshiyuki Sakurai, Mariko Shimada, Yuko Iwashita, Naoki Shibuya, Yoshihiro Akita, Haruna Miyashita, Yuki Maruyama, Masayuki Saruta

**Affiliations:** Division of Gastroenterology and Hepatology, Department of Internal Medicine, The Jikei University School of Medicine, 3-25-8 Nishi-shinbashi, Minato-ku, Tokyo, 105-8461 Japan

**Keywords:** Budesonide foam, Bowel frequency (night), Urgent defecation, 5-ASA topical treatment

## Abstract

**Introduction:**

Patients with ulcerative colitis (UC) are known to have a significantly poor quality of life due to bowel frequency (night) and urgent defecation. Budesonide foam is a topical medication that was approved in Japan in 2017 for the treatment of UC. However, its efficacy in the treatment of bowel frequency (night) or urgent defecation is unknown. This study aimed to explore the efficacy of budesonide foam for the alleviation of these symptoms.

**Methods:**

UC patients who received budesonide foam between December 2017 and January 2020 at the Jikei University School of Medicine in Tokyo were enrolled. The simple clinical colitis activity index (SCCAI) was evaluated at the start of budesonide foam treatment and 2 and 6 weeks later in patients who initially scored ≥ 1 for bowel frequency (night) and urgent defecation, respectively. We also studied the effect of budesonide foam on remaining symptoms in patients who had used 5-aminosalicylic acid (5-ASA) topical treatment, those with SCCAI ≥ 3, and those in remission with residual symptoms (SCCAI 1 or 2).

**Results:**

Of the 233 enrolled patients, 102 were eligible for the study. In 36 patients with bowel frequency (night) treated with budesonide foam were significantly effective, score in SCCAI decreased from 1.17 ± 0.45 at baseline to 0.53 ± 0.61 at week 2 (*p* < 0.0001) and 0.17 ± 0.38 at week 6 (*p* < 0.0001). In 45 patients with urgent defecation score in SCCAI decreased significantly from 1.33 ± 0.52 at baseline to 0.44 ± 0.59 at week 2 (*p* < 0.0001) and 0.22 ± 0.40 at week 6 (*p* < 0.0001). Of 22 patients who switched from topical 5-ASA administration to budesonide foam, nine at week 2 (41%) and 11 (50%) at week 6 were improved with no symptoms, and there were no cases of worsened symptoms. No severe side effects associated with budesonide foam were observed.

**Conclusion:**

Budesonide foam administration significantly improves both bowel frequency (night) and urgent defecation-related UC activity and is also effective for the patients who were refractory to topical 5-ASA administration.

## Introduction

Ulcerative colitis (UC) is an inflammatory bowel disease caused by diffuse inflammation of the colonic mucosa with erosions or ulcers; the pathogenesis is still unknown. Inflammation starts from the rectum and extends to the entire large intestine. The major symptoms of UC are diarrhea, bloody stools, abdominal pain [1–3], bowel frequency (night) and urgent defecation. Urgent defecation has been identified as an important symptom in many studies [4–7], is emphasized in the United States guidelines, and is used as a success index for diagnosis and treatment [8]. An online survey of Japanese UC patients confirmed that urgent defecation correlates with a decrease in the patient's quality of life (QOL) [4]. Although there are only a few reports of bowel frequency (night) caused by UC, this symptom induces sleep disturbance and significantly reduces QOL [9, 10]. Since these symptoms are directly related to the QOL, it is essential to alleviate them immediately as persistent chronic inflammation can increase the risk of colorectal cancer and should be treated appropriately.

Oral 5-aminosalicylic acid (5-ASA) preparations are used to treat mild to moderate UC. Still, often the dose is not sufficient to reach the distal colon and rectum, resulting in residual rectal and sigmoid colonic inflammation and their related symptoms [11, 12]. In such cases, 5-ASA topical treatments such as enema or suppositories are useful for distal residual inflammation [13, 14]. Topical therapy consisting of 5-ASA or steroid suppositories and enemas can be used in combination with orally administered drugs [15–17]. Budesonide foam approved in Japan in 2017 can be highly effective by spraying a sufficient amount of the drug on the distal colon [18–20]. For six weeks, the phase III study performed in Japan showed that budesonide foam improved rectal bleeding subscores and endoscopic scores and induced remission [21]. However, there have been no studies on the effect of budesonide foam on urgency and bowel frequency (night), which are directly linked to the QOL. Budesonide foam was significantly effective in the studies compared with topical 5-ASA [20, 22]; on the contrary, comparison studies with topical 5-ASA primarily focusing on the prolonged clinical symptoms have not been performed.

Therefore, this study aimed to examine the efficacy of budesonide foam for treating bowel frequency (night) and urgency defecation in UC patients and its effectiveness when switched to inpatients that showed lower efficiency with topical 5-ASA administration.

## Materials and methods

A retrospective study was conducted at the Jikei University School of Medicine in Tokyo. We investigated the electronic medical records of 233 patients with UC who started budesonide foam treatment between December 2017 and January 2020 at our hospital. We obtained information on the patient background (sex, age, disease type, and duration of illness), Mayo endoscopic subscore before budesonide foam administration, coexisting treatments, and overall scores for each item of the Simple Clinical Colitis Activity Index (SCCAI) for UC before and 2 and 6 weeks after budesonide foam administration. We evaluated patients who have treated it for 6 consecutive weeks alone in this study, and exclude patients who used it on demand. We also noted the presence and type of adverse events during this study. Patients whose symptoms could not be quantified 2 and 6 weeks after the start of budesonide foam and patients who were administered a new induction drug during this period were excluded from the study.

We studied the presence or absence of changes in the scores of bowel frequency (night) and urgent defecation, which are sub-items of the SCCAI, at weeks 2 and 6 after budesonide foam administration. We also studied the presence or absence of changes in the SCCAI score at baseline, week 2, and week 6 after changing to budesonide foam to improve symptoms in UC patients who had prolonged symptoms and had used topical 5-ASA administration for more than four weeks. Similarly, the efficacy rate (induction rate of remission) at weeks 2 and 6 after administration was studied for patients with active UC treated with budesonide foam. Finally, we investigated the symptom disappearance rate by budesonide foam at weeks 2 and 6 in patients in clinical remission at baseline but had residual symptoms (SCCAI 1 or 2). SCCAI 0, ≤ 2, and ≥ 3 were defined as SCCAI 0 clinical remission (symptom free), clinical remission, and active UC, respectively.

### Statistical methods

The comparison of SCCAI scores and sub-items of SCCAI before and after the budesonide foam administration were analyzed using the Wilcoxon signed rank-sum test. The McNemar test was performed to compare the improvement and remission rates in each SCCAI evaluation period. Statistical significance was set at *p* < 0.05. Data are expressed as mean ± standard deviation or median with interquartile range. SPSS and STATA version 15 were used for statistical analysis**.**

## Results

### Patient characteristics

**Fig. 1:**
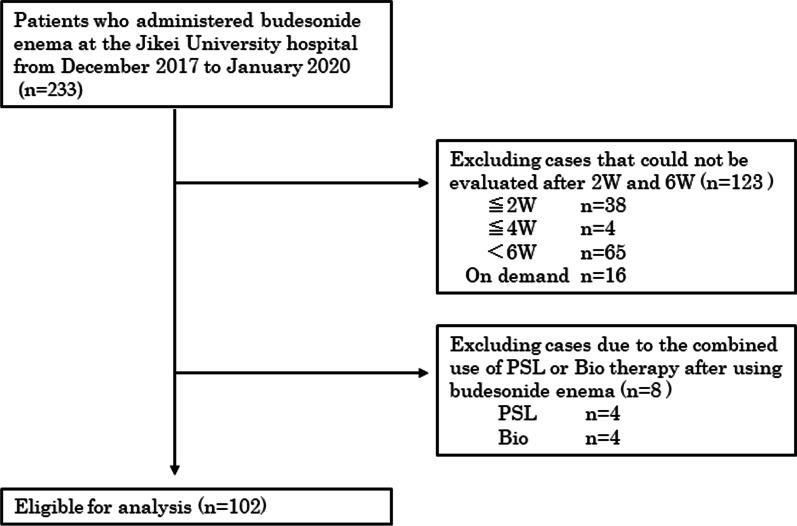
Flow diagram of subjects

Of the 233 patients with UC who started budesonide foam administration, 102 were eligible for the study shown in Fig. 1. The patient backgrounds are shown in Table 1. The average age of the patients was 42.8 ± 14.2 years, and there were 38 males and 64 females. Pancolitis occurred in 49 cases (48%), left-sided colitis in 37 cases (36%), and proctitis in 16 cases (16%). Patients treated with immunosuppressive drugs such as tacrolimus and cyclosporine, and cytapheresis were not included.

**Table 1:** Baseline demographics and clinical parameters

Variable	Category	
Age, mean (years)		42.8 ± 14.2
Sex, *n* (%)	Female	64 (62.7)
	Male	38 (37.3)
Disease type, *n* (%)	Pancolitis	49 (48.0)
	Left-sided colitis	37 (36.3)
	Proctitis	16 (15.7)
	(Distal type)	40 (39.2)
Mayo endoscopic subscore, *n* (%)	Score 0	15 (14.7)
	Score 1	43 (42.2)
	Score 2	29 (28.4)
	Score 3	5 (4.9)
Simple clinical colitis activity index	Score 0	3 (2.9)
(SCCAI), *n* (%)	Score 1–2	29 (28.4)
	Score ≥ 3	70 (68.6)
Simple clinical colitis activity index	0 week	4.0 ± 2.6
(SCCAI), mean	2 weeks	1.9 ± 2.0
	6 weeks	1.1 ± 1.7
Bowel frequency (night) score, *n* (%)		36 (35.3)
Defecation urgency score, *n* (%)		45 (44.1)
Both bowel frequency (night) and		27 (26.4)
defecation urgency score, *n* (%)		
Concomitant drug, *n* (%)	5-ASA suppositories	22 (21.5)
	5-ASA enemas	3 (2.9)
	Prednisolone suppositories	1 (1.0)
	Prednisolone enemas	6 (5.8)
Immunomodulators, *n* (%)		11 (10.7)
Biologics, *n* (%)	Infliximab	5 (4.9)
	Golimumab	3 (2.9)
	Adalimumab	2 (1.9)
	Vedolizumab	1 (1.0)
CRP at start of the observation,		0.8 ± 1.6
mean (mg/dl)		

### Treatment efficacy of budesonide foam of UC patients with bowel frequency (night) / urgency defecation

**Fig. 2:**
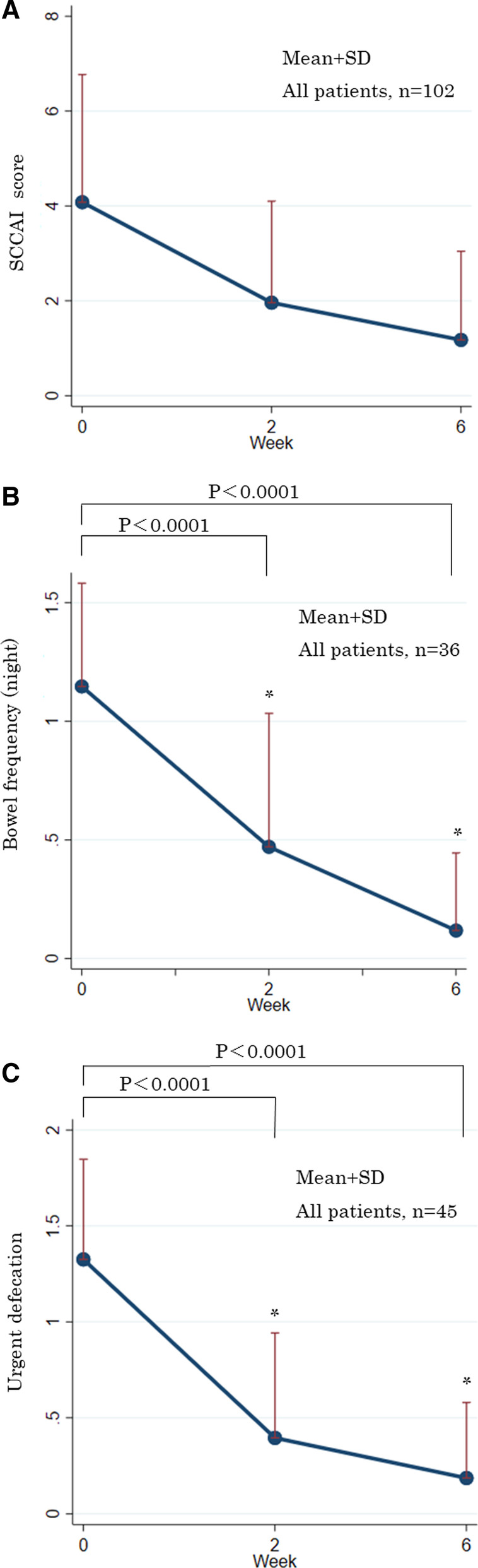
**A** Changes in the Simple Clinical Colitis Activity Index (SCCAI) after budesonide foam administration in patients with mild-to-moderate ulcerative colitis. Numbers in the graph are the number of patients evaluated at each visit. Markers in the graph represent the mean + standard deviation for each score. **B**. Changes in bowel frequency (night) score after budesonide foam administration in patients with mild-to-moderate ulcerative colitis. Numbers in the graph are the number of patients evaluated at each visit. Markers in the graph represent the mean + standard deviation for each score. * *p* < 0.001 versus baseline (week 0) scores. **C** Changes in urgent defecation score after budesonide foam administration in patients with mild-to-moderate ulcerative colitis. Numbers in the graph are the number of patients evaluated at each visit. Markers in the graph represent the mean + standard deviation for each score. * *p* < 0.001 versus baseline (week 0) scores

The mean SCCAI score decreased significantly from 4.01 ± 2.66 at baseline to 1.86 ± 2.03 at week 2 and 1.10 ± 1.76 at week 6 shown in Fig. 2A. In 36 of the 102 eligible subjects with bowel frequency (night) score in SCCAI decreased significantly from 1.17 ± 0.45 at baseline to 0.53 ± 0.61 (*p* < 0.0001) at week 2 and 0.17 ± 0.38 at week 6 (*p* < 0.0001) shown in Fig. 2 B. In 45 cases with urgent defecation score in SCCAI decreased significantly from 1.33 ± 0.52 at baseline to 0.44 ± 0.59 at week 2 (*p* < 0.0001) and 0.22 ± 0.40 at week 6 (*p* < 0.0001) shown in Fig. 2 C. There were no cases of worsening score during this study period.

### Clinical benefits of UC patients that switched to budesonide foam from topical 5-ASA preparation

**Fig. 3:**
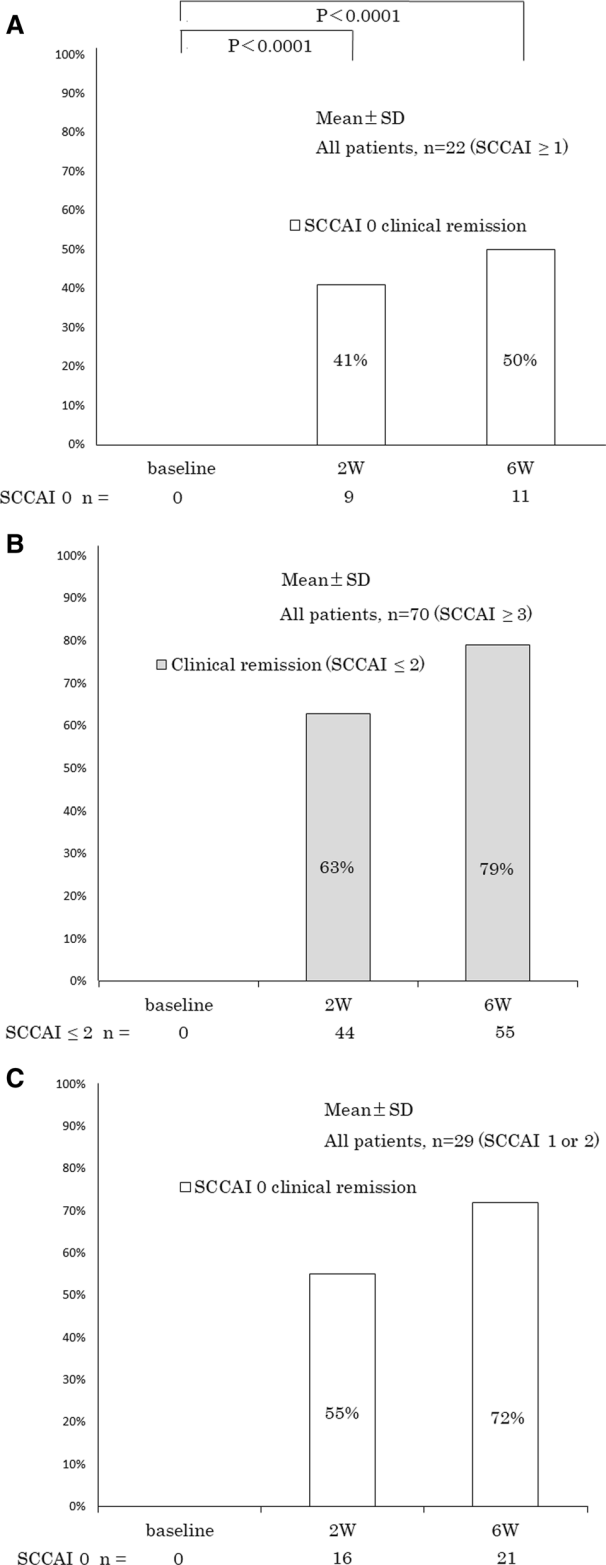
**A** Changes in SCCAI 0 clinical remission rates at baseline, week 2, and week 6 of patients who switched to budesonide foam from topical 5-ASA preparation because of residual symptoms (SCCAI ≥ 1). **B** Changes in clinical remission rates (SCCAI ≤ 2) at baseline, week 2, and week 6 after budesonide foam administration. **C**. Changes in SCCAI 0 clinical remission rates at baseline, week 2, and week 6 after budesonide foam administration for the patients with residual symptoms in clinical remission (SCCAI 1 or 2)

Of the 22 patients who switched from topical 5-ASA preparation to budesonide foam to improve symptoms, 12 patients (SCCAI ≥ 3) were included due to unsuccessful induction treatments of remission. The remaining ten patients (SCCAI 1 or 2) were in clinical remission score but had residual clinical symptoms. The number of patients without any symptoms significantly increased at week 2 (*n* = 9, 41%, *p* < 0.0001) and week 6 (*n* = 11, 50%, *p* < 0.0001) shown in Fig. 3A.

### *Clinical improvement of budesonide foam in patients with active UC (SCCAI* ≥ *3)*

Seventy of the 102 target patients exhibited UC clinical activity (SCCAI ≥ 3) at the start of budesonide foam. Of these patients, 63% (*n* = 44) achieved clinical remission (SCCAI ≤ 2) at week 2 and 79% (*n* = 55) at week 6 shown in Fig. 3 B.

### Clinical efficacy of budesonide foam for UC patients with residual clinical symptoms (SCCAI 1 or 2)

Of the 29 patients who had residual symptoms even in clinical remission (SCCAI 1 or 2) at baseline, 55% (*n* = 16) had complete disappearance (SCCAI 0) of symptoms at week 2 and 72% (*n* = 21) at week 6 shown in Fig. 3 C. Among these, three patients underwent colonoscopy both before and after budesonide foam administration, and all three patients were induced to mucosal healing with Mayo endoscopic subscore 0.

### Reports of adverse events of budesonide administration

Adverse events such as opportunistic infections, Cushing's syndrome, osteoporosis, rectal injury, diabetes mellitus, cataracts, glaucoma and neuropathy were not observed in any subject during this study.

## Discussion

This study is the first paper on the budesonide foam rapidly improves the most prominent UC symptoms, such as bowel frequency (night) and urgent defecation. According to previous reports about factors influencing the QOL of UC patients in Japan, the daily life of 43.5% of these patients was "significantly affected" by UC symptoms [4]. Similarly, bowel frequency (night) is also closely related to the QOL of UC patients. It has been suggested that the frequency of stools and rectal bleeding should not be considered alone in determining the QOL of these patients [910]. Furthermore, the QOL was not investigated in detail in the study; we confirmed that bowel frequency (night) and urgent defecation improved significantly at week 2 after budesonide foam administration, indicating that the QOL was also improved.

Our findings confirm the use of budesonide foam as an excellent alternative treatment that can significantly improve the QOL in UC patients. In UC, inflammation occurs from the rectum just above the anus; therefore, eliminating rectal inflammation is crucial for the complete disappearance of symptoms. In the initial treatment of UC, 5-ASA preparations are usually administered regardless of disease type to induce remission, but there may be minor residual inflammation and symptoms. Our results show that budesonide foam can also eliminate residual inflammation in such cases. Half of the patients utilizing a 5-ASA topical preparation was ineffective, but when substituted with budesonide foam, the symptoms disappeared in 6 weeks, and no worsening cases were observed. Thus, if 5-ASA topical preparations do not induce remission, budesonide foam could be a viable treatment option before systemic steroid administration.

Improvement of the rectal bleeding and urgent defecation two weeks after budesonide foam administration is associated with complete mucosal remission six weeks after treatment [23, 24]. However, only the rectal bleeding and urgent defecation were evaluated, and the response rates for all other clinical symptom scores were unclear; similar results were obtained in the present study. Moreover, the remission rate increased by 16% at week 6 compared to week 2. Thus, even if remission is not achieved at week 2 after budesonide foam administration, it is better to continue the treatment until week 6.

UC patients in clinical remission with a residual SCCAI symptom score of 1 or 2 are commonly observed in routine clinical practice. This study revealed that budesonide foam was effective even for mild residual symptoms such as urgent defecation, mild rectal bleeding, and bowel frequency (night). There are no research reports that present a correlation between SCCAI scores ≤ 2 and QOL. However, these symptoms do have the potential to affect the QOL of patients with UC. The high rate of symptom alleviation observed in our study (more than 50% of the patients at 2 weeks and 70% at 6 weeks) suggests that this formulation is highly beneficial for improving the QOL of these patients. Even though budesonide foam is easier to use than conventional topical preparations, the difficulty of inserting budesonide foam twice a day is a disadvantage for the patient's QOL. Nevertheless, considering that the symptoms can be improved by using budesonide foam for a certain period, it is considered that the overall merit of its use is higher than non-use.

No noticeable side effects were reported in any of the cases in this study. Also, serum cortisol values were not measured in this study, and there was no change in the cortisol value in a previous report [25]. Thus, budesonide foam can be selected as an optional treatment without any safety concerns.

The main limitation of this study is that it was a single-center, retrospective study. In particular, the use of budesonide foam was not based on specific criteria and rather reflects the intentions of the attending physician or patient; thus, selection bias was not excluded. Second, the SCCAI is not a definite objective evaluation index because it is easy for patients to have their own subjective feelings about their symptoms. However, improvement was seen with the SCCAI before and after treatment in the same patient; hence, a certain degree of objectivity was achieved. Additionally, a few patients who received new treatment during the six weeks were excluded from the study, and it is possible that budesonide foam was not effective in those patients. Finally, the adherence rate of budesonide foam administration is uncertain because it was not confirmed the administration rate of the prescribed medication.

## Conclusion

Budesonide foam significantly improves the scores of bowel frequency (night) and urgent defecation among patients with UC symptoms. It is also effective in refractory cases changed from a 5-ASA preparation and has the ability to induce instant remission. Furthermore, the remission rate was higher at week 6 than that of week 2 after budesonide foam administration, indicating the need to continue for six weeks.

## Data Availability

All data needed to evaluate the conclusions in the paper are presented in the paper. Additional data related to this paper may be requested from the authors.
