# Correction: Bowel frequency (night) and urgent defecation are improved by budesonide foam in patients with ulcerative colitis: a retrospective observational study

**DOI:** 10.1186/s12876-022-02445-0

**Published:** 2022-08-25

**Authors:** Ryosuke Miyazaki, Toshiyuki Sakurai, Mariko Shimada, Yuko Iwashita, Naoki Shibuya, Yoshihiro Akita, Haruna Miyashita, Yuki Maruyama, Masayuki Saruta

**Affiliations:** Division of Gastroenterology and Hepatology, Department of Internal Medicine, The Jikei University School of Medicine, 3-25-8 Nishi-shinbashi, Minato-ku, Tokyo, 105-8461 Japan

## Correction to: BMC Gastroenterology (2022) 22:310 10.1186/s12876-022-02388-6

After publication of this article [1], the authors reported that the ‘Competing interests’ section was incomplete and should have been:

M. Saruta has received consulting and lecture fees from Abbvie GK., Janssen Pharmaceutical K.K., Mitsubishi Tanabe Pharma Co., Ltd., Takeda Pharmaceutical Co., Ltd, EA Pharma Co., Ltd., and Gilead K.K; and research grants from EA Pharma Co., Ltd., EP-CRSU Co., Ltd., Mitsubishi Tanabe Pharma, Mochida Pharmaceutical Co., Ltd., and Zeria Pharmaceutical Co., Ltd.

The other authors have no conflicts of interest related to this article.

The original article [1] has been corrected.
